# Association of High-Sensitivity Cardiac Troponin I, Platelet Counts, and Total Bilirubin/Albumin Ratio with the Severity of Neonatal Hyperbilirubinemia

**DOI:** 10.33549/physiolres.935562

**Published:** 2025-08-01

**Authors:** Fang LU, Guangyao CHEN, Ju YUAN, Jun DENG

**Affiliations:** 1 Department of Pediatrics, JingMen People’s Hospital, Jingchu University of Technology Affiliated JingMen People’s Hospital, Jingmen, Hubei Province, China; 2 Department of Neonatal, JingMen People’s Hospital, Jingchu University of Technology Affiliated JingMen People’s Hospital, Jingmen, Hubei Province, China; 3 Department of Neonatal, Xiantao Maternal and Child Healthcare Hospital, Xiantao, Hubei province, China

**Keywords:** Bilirubin/albumin ratio, High-sensitivity cardiac troponin I, Jaundice severity, Neonatal hyperbilirubinemia, Platelets

## Abstract

This study aimed to investigate the association between serum high-sensitivity cardiac troponin I (hs-cTnI) concentrations, platelet counts, and the severity of neonatal hyperbilirubinemia (NHB), as well as their correlation with the total bilirubin/albumin (B/A) ratio. A total of 82 neonates diagnosed with NHB and treated at JingMen People’s Hospital of Hubei Province, China, between September 2023 and December 2023 were enrolled. Participants were categorized into mild and severe NHB groups based on total bilirubin levels. Serum hs-cTnI concentrations, platelet counts, and total bile acid levels were compared between groups. Correlations among hs-cTnI, platelet counts, and the B/A ratio were assessed. Neonates in the severe NHB group exhibited significantly higher hs-cTnI concentrations (median 64.1 [31.7, 92.1] ng/mL) compared with those in the mild NHB group (median 41.9 [17.55, 60.4] ng/mL; *p*=0.015). The B/A ratio was also significantly higher in the severe group (10.5 [10.0, 10.9] vs. 8.4 [7.9, 8.9]; *p*<0.001). No significant differences were observed between groups regarding platelet counts or total bile acid levels. Additionally, neither platelet count nor the B/A ratio demonstrated a significant correlation with hs-cTnI concentrations. Neonates with severe hyperbilirubinemia exhibited significantly elevated hs-cTnI concentrations and B/A ratios. However, hs-cTnI levels were not significantly influenced by platelet counts or the B/A ratio.

## Introduction

Neonatal hyperbilirubinemia (NHB) is a common condition, affecting approximately 49.1 % of hospitalized neonates in China. Without timely intervention, NHB can result in neurological sequelae, including hearing loss, as well as intellectual and motor developmental disorders [1]. Biliverdin, a byproduct of heme metabolism, is reduced to free bilirubin (Bf) by biliverdin reductase. Less than 1 % of free bilirubin remains unbound to albumin. While low concentrations of unconjugated bilirubin (UCB) may exert antioxidative effects upon entering cells, elevated levels are associated with pro-oxidative and cytotoxic properties, with cytotoxic thresholds varying among different cell types [2]. Autopsy findings in neonates with hyper-bilirubinemia have reported widespread bilirubin deposition in vital organs, including the liver, kidneys, heart, adrenal glands, lungs, and brain [3].

As free bilirubin cannot be directly measured clinically and correlates positively with circulating unconjugated bilirubin levels [4], the bilirubin-to- albumin (B/A) ratio is used as an important indicator for assessing bilirubin toxicity. Elevated bilirubin concentrations may induce myocardial injury by disrupting mitochondrial oxidative metabolism, competing for albumin binding, inhibiting myocardial ATP activity, and leading to toxin accumulation. The immature neonatal myocardium, characterized by limited energy reserves and reduced compensatory capacity, is particularly susceptible to damage.

Cardiac troponin I (cTnI), expressed exclusively in atrial and ventricular myocardium, contains an immunologically distinct N-terminal amino acid sequence [5] and is not present in skeletal muscle tissue [6]. Compared to creatine kinase-MB (CK-MB), cTnI offers superior sensitivity and specificity for detecting myocardial injury, establishing it as the clinical gold standard [7]. Traditional cTnI assays, with a detection limit of 500 ng/L, primarily detect significant myocardial damage, whereas high-sensitivity cardiac troponin I (hs-cTnI) assays can detect concentrations as low as 5 ng/L, enabling earlier detection [6].

Recent studies have reported that neonates with a B/A ratio>9.48 are at higher risk for acute bilirubin encephalopathy [8]. Furthermore, cTnI has been identified as a potential diagnostic and prognostic marker for myocardial injury in hemolytic hyperbilirubinemia [9]. The relationship between hs-cTnI levels and the severity of neonatal hyperbilirubinemia has also been explored [10], although whether the B/A ratio influences hs-cTnI concentrations in this population remains unclear.

The present study focused on neonates with jaundice admitted to JingMen People’s Hospital of Hubei Province, China, between September 2023 and December 2023. The aim was to analyze potential differences in serum hs-cTnI concentrations, platelet counts, and total bile acid levels among neonates with varying degrees of hyperbilirubinemia, and to investigate whether hs-cTnI levels are influenced by platelet counts and the B/A ratio.

## Materials and Methods

### Study sample

Neonates diagnosed with NHB and admitted to the Department of Neonatology at JingMen People’s Hospital of Hubei Province, China, between September 2023 and December 2023, were enrolled in this study.

Inclusion criteria were as follows: (1) gestational age ≥ 37 weeks; (2) birth weight ≥ 2.5 kg; (3) postnatal age ≥ 3 days; and (4) availability of complete clinical data.

Exclusion criteria included: (1) low Apgar score ≤ 7 after five minutes; (2) preterm birth, small-for-gestational-age status, or low birth weight; (3) presence of infection, pneumonia, or other primary conditions associated with myocardial injury; (4) hemolytic disease, cephalohematoma, biliary malformation or obstruction, or direct bilirubin concentration>34.2 μmol/L; (5) severe congenital anomalies or inherited metabolic disorders; and (6) incomplete clinical data.

### Observation indicators

Clinical data were extracted from hospital records, including gestational age, mode of delivery, birth weight, neonatal age (days), sex, total serum bilirubin (TSB), albumin (Alb), platelet count (PLT), hs-cTnI, and total bile acid concentrations. The B/A ratio was calculated accordingly.

Hs-cTnI concentrations were measured using an enzyme-linked immunofluorescence assay performed on the VIDAS® automated fluorescence analyzer (bioMérieux SA, France). The VIDAS® High-Sensitivity Troponin I (TNHS) kit, with a detection range of 4.9–40,000 ng/L, was utilized.

### Statistical analysis

All data analyses were performed using SPSS software, version 25.0 (IBM Corp., Armonk, NY, USA).

Continuous variables with a normal distribution were expressed as mean ± standard deviation (SD) and compared between groups using the independent-samples Student’s *t*-test. Continuous variables with a non-normal distribution were expressed as median (interquartile range [IQR]) and compared using the Mann–Whitney U test.

Categorical variables were expressed as frequencies and percentages (n [%]) and compared between groups using the chi-squared test or Fisher’s exact test, as appropriate. Logistic regression analysis was conducted to evaluate the influence of platelet counts and B/A ratio on hs-cTnI concentrations.

A two-sided *p*-value <0.05 was considered indicative of statistical significance.

## Results

A total of 82 neonates were enrolled in the study and categorized into two groups based on TSB levels: the mild NHB group (TSB<342 μmol/L; n=51) and the severe NHB group (TSB ≥ 342 μmol/L; n=31). A significant difference in age was observed between the two groups (*p*=0.018); however, no significant differences were found regarding gestational age, sex, birth weight, or mode of delivery (*p*>0.05). Detailed demographic and clinical characteristics are presented in Table 1.

In the mild NHB group, the median hs-cTnI concentration was 39.90 (16.38–58.70) ng/mL, and the median B/A ratio was 8.43 (7.95–8.92). In the severe NHB group, the median hs-cTnI concentration (64.10 [31.70–92.10] ng/mL) and the median B/A ratio (10.48 [9.99–10.89]) were significantly higher than those in the mild group (*p*=0.008 and *p*<0.001, respectively). No significant differences were observed between groups in platelet counts or total bile acid levels (*p*>0.05). These results are summarized in Table 2.

**Fig. 1:**
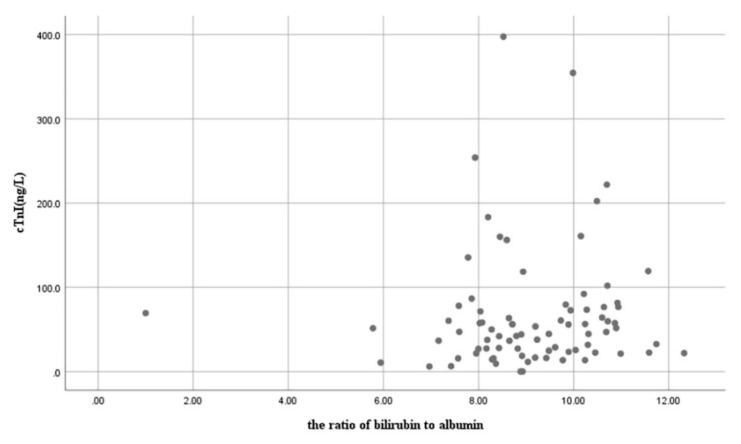
Correlation between B/A ratio and hs-cTnI concentrations.

**Fig. 2:**
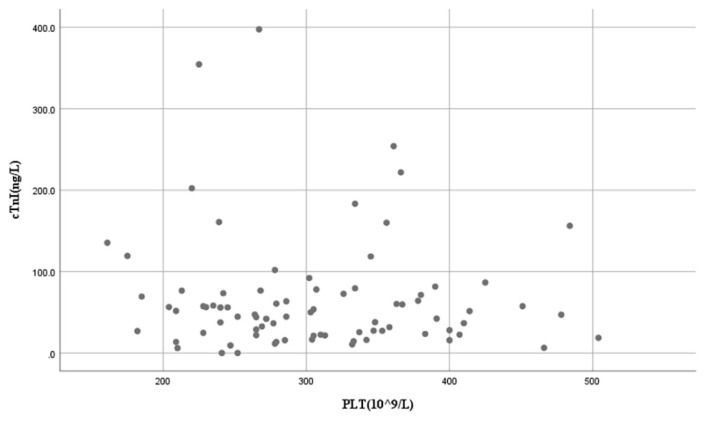
Correlation between platelet count and hs-cTnI concentrations.

Spearman’s correlation analysis demonstrated a weak but statistically significant positive correlation between TSB levels and hs-cTnI concentrations (*r*=0.25, *p*=0.025) (Fig. 1). Linear regression analysis evaluating the effects of platelet count and B/A ratio on hs-cTnI concentrations yielded *p*-values of 0.511 and 0.650, respectively, indicating no significant linear associations (Fig. 2).

## Discussion

In this study, a significant difference in neonatal age was observed between the mild and severe NHB groups (*p*=0.018). Although healthy neonates typically exhibit higher serum cTnI levels at birth compared to adults, these levels generally decline to near-adult concentrations within the first three days of life. Neonatal cTnI concentrations are not significantly influenced by maternal factors, sex, or gestational age [11]. As all neonates enrolled in the present study were over three days old, the observed age difference between groups was unlikely to have affected the hs-cTnI results.

A key finding was that neonates with severe NHB exhibited significantly higher hs-cTnI concentrations compared to those in the mild group. Previous studies have reported that even moderate elevations in bilirubin levels (205.2–256.5 μmol/L) can result in increased cTnI concentrations, indicative of myocardial injury. Further elevations in bilirubin levels have been associated with significant increases in both cTnI and CK-MB, suggesting progressive myocardial cell injury [12].

Unconjugated bilirubin (UCB), acting as a pro-apoptotic mediator, has been shown to inhibit cell proliferation by inducing DNA fragmentation, promoting cytochrome c release, and activating caspase-3. High concentrations of unbound UCB impact mitochondria by downregulating B-cell lymphoma 2 (Bcl-2) expression, enhancing Bcl-2-associated X protein (Bax) activity, and triggering the release of apoptotic factors, thereby upregulating caspase-3 [13]. Elevated bilirubin concentrations in neonates are thus associated with more severe myocardial damage [14]. Consistent with the present findings, Liu *et al*. reported that serum TSB levels>342 μmol/L were associated with the highest cTnI concentrations in neonates [15].

In the current study, a weak but statistically significant positive correlation was observed between TSB and hs-cTnI concentrations (*r*=0.25, *p*=0.025), while no correlation was found between the B/A ratio and hs-cTnI levels. Bai *et al*. similarly reported that hyperbilirubinemia had a more pronounced effect on myocardial cTnI concentrations compared to infection or prematurity; however, cTnI levels did not increase proportionally with bilirubin levels, and no significant difference was observed between hemolytic and non-hemolytic hyperbilirubinemia in this regard [16].

Experimental findings from a neonatal rat model of hyperbilirubinemia-induced myocardial apoptosis revealed that marked increases in serum cTnI accompanied elevated TSB concentrations, with myocardial apoptosis demonstrating concentration- and time-dependent characteristics. Nevertheless, pathological changes in myocardial tissue structure were not observed, suggesting that hyperbilirubinemia induces mild myocardial injury without proportional structural damage [13]. This may provide a mechanistic explanation for the weak correlation observed in the present study.

Additionally, platelet counts were not found to influence hs-cTnI concentrations in this cohort. Bilirubin toxicity has been shown to stimulate the expression of cytokines, such as macrophage-granulocyte colony-stimulating factor, promoting megakaryocyte maturation and resulting in secondary thrombocytosis. Moreover, pathogenic factors underlying hyperbilirubinemia may contribute to elevated platelet counts to a certain extent.

Animal studies have demonstrated that platelet-rich plasma can reduce cTnI expression in myocardial cells following ischemia-reperfusion injury by promoting AMP-activated protein kinase phosphorylation and modulating macrophage activity, thereby exerting cardioprotective effects [17]. However, increased platelet counts may also promote local thrombosis, obstructing hepatic microvasculature, impairing bilirubin clearance and sustaining elevated plasma bilirubin concentrations, which could diminish the cardioprotective benefits of platelets.

Several limitations of this study should be acknowledged. First, the retrospective design may have introduced selection and information bias. Second, the relatively small sample size and single-center setting may limit the generalizability of the findings. Future research should involve larger, multicenter cohorts or meta-analyses to strengthen the evidence base and validate these preliminary findings.

## Conclusion

This study demonstrated that neonates with severe NHB exhibited significantly higher hs-cTnI concentrations and elevated B/A ratios compared to those with mild NHB. However, hs-cTnI concentrations were not significantly influenced by platelet counts or the B/A ratio. Limitations of this study include the absence of neonates with extremely severe hyperbilirubinemia, which may limit the scope of the comparisons. Future large-sample, multicenter studies encompassing a broader range of disease severity are warranted to further clarify the relationship between hyperbilirubinemia and myocardial injury in neonates.

## Figures and Tables

**Table 1:** Comparison of Baseline Characteristics Between the Mild NHB and Severe NHB Groups

	Mild NHB group (n=51)	Severe NHB group (n=31)	t	Z	χ^2^	p
*Gestational age (weeks)*	38.7±0.8	38.4±0.9	1.295			0.199
*Neonatal age (days)*	4(3,5)	5(4,6)		−2.363		0.018
*Sex*					0.204	0.651
*Male (n)*	24(47.1 %)	13(41.9 %)				
*Female (n)*	27(52.9 %)	18(58.1 %)				
*Birth weight (kg)*	3.21±0.37	3.29±0.42	−0.87			0.387
*Mode of delivery*					0.299	0.585
*Cesarean section (n)*	42(82.4 %)	24(77.4 %)				
*Vaginal delivery (n)*	9(17.6 %)	7(22.6 %)				

**Table 2:** Comparison of Observational Indicators Between the Mild NHB and Severe NHB Groups

	Mild NHB group	Severe NHB group	Z	*t*	*p*
*hs-cTnI(ng/L)*	39.90 (16.38,58.70)	64.10 (31.70,92.10)	−2.643		0.08
*B/A*	8.43 (7.95,8.92)	10.48 (9.99,10.89)	−6.656		0.00
*PLT(10^9/L)*	302.55±78.27	313.71±85.02		0.338	0.736
*Total bile acids (mol/L)*	14.65 (9.75,23.83)	14.50 (8.90,19.70)	−0.311		0.756
